# Physiological and Cellular Ultrastructural Responses of *Sesuvium portulacastrum* under Cd Stress Grown Hydroponically

**DOI:** 10.3390/plants12193381

**Published:** 2023-09-25

**Authors:** Mohammad Mazbah Uddin, Zhenfang Chen, Fuliu Xu, Lingfeng Huang

**Affiliations:** 1 Key Laboratory of the Ministry of Education for Earth Surface Processes, College of Urban & Environmental Sciences, Peking University, Beijing 100871, China; mazbah_uddin88@outlook.com; 2 Key Laboratory of the Ministry of Education for Coastal and Wetland Ecosystems, College of the Environment and Ecology, Xiamen University, Xiamen 361005, China; chenzhenfang202308@163.com

**Keywords:** heavy metals, cadmium, halophyte, physiology, antioxidants

## Abstract

This study aimed to investigate the physiological and cellular mechanisms of *Sesuvium portulacastrum* under heavy metal stress to evaluate possible tolerance and adaptation mechanisms in a metal-polluted environment. The physiological and cellular ultrastructural responses of *S. portulacastrum* were studied hydroponically under exposure to a range of cadmium (Cd) concentrations (50 µM to 600 µM) for 28 days. The activity of antioxidant enzymes like catalase (CAT), superoxide dismutase (SOD), and peroxidase (POD), changes in chlorophyll, and cellular ultrastructural content were examined. There was no significant difference in chlorophyll content in the leaf under the stress of 300 μM, but 400 μM and 600 μM Cd stress showed significantly decreased chlorophyll content. The SOD activity indicates an increase under the Cd stress of 100 μM for leaves, 300 μM for stems, and 50 μM for roots; after that, the SOD activity gradually decreased with increasing Cd concentrations. But POD activity was considerably increased with increasing Cd stress. CAT activity showed a gradual increase in concentrations until 300 μM of Cd stress and then decreased sharply in roots, stems, and leaf tissues. Cd stress had a considerable impact on the structure of the roots, stems, and leaves cells, such as distorted and thinner cell walls and the deformation of chloroplasts, mitochondria, and other organelles. Therefore, the increased number of nucleolus in the cell nucleus suggests that cells may be able to maintain their protein synthesis in a stressful environment. This study concludes that SOD is the dominant antioxidant enzyme activity during low Cd toxicity (<100 μM), while POD is the dominant enzyme activity during higher Cd toxicity (>100 μM).

## 1. Introduction

Cadmium (Cd) is a non-essential and noxious metal in environmental mediums. The intensified blooming of chemical, fertilizer, pesticide, marine shipping, and petrochemical industries globally is increasing the discharge of chemical pollutants, including Cd to the lagoons, rivers, lakes, and oceans [1,2,3]. Due to these chemical pollutants, the biological quality and ecological environment of whole marine and terrestrial ecosystems are damaged, intensifying the degree of pollution [4,5]. Moreover, cadmium can accumulate in sediments, plants, and aquatic organisms and ultimately be transported to the food web, which is causing human health issues [6,7,8,9]. In current years, cadmium is considered to be the main pollutant in China’s agricultural land and coastal areas due to its industrial revolution over the last few decades [10]. Cadmium can produce carcinogenicity, mutagenicity, and teratogenic effects on human health and serious problems in aquatic ecosystems such as bioaccumulation and biomagnification in the food web [11,12]. 

Cadmium can be taken through plant roots and transferred to different tissues of the plant [13]. Higher accumulations of Cd in plants have negative effects such as chlorosis, inhabitation of plant growth, nutrient absorption and metabolism, nutrient imbalances, cellular ultrastructure deformation and destruction of organelles, and accumulation of “reactive oxygen species” (ROS), which all trigger plants to die off [12,13,14,15,16]. Higher Cd stress or concentration in plants can enhance stress, such as the accumulation of “reactive oxygen species” (ROS) in plants, which is a major evidence of Cd poisoning [12]. To reduce oxidative damage, plants can enhance the antioxidant response system, which can maintain redox homeostasis and the clearance of ROS [17]. The plant antioxidant response system consists of non-enzymatic and enzymatic activities. The non-enzymatic activities are proline, carotenoids, phenols, flavonoids, etc. [12]. Enzymatic antioxidant activities include SOD, POD, CAT, etc. [12]. Several studies claimed that under the stress of Cd, SOD, POD, and CAT activities considerably increased [12,18,19]. In perennial Ryegrass plants, activities of SOD, POD, and CAT increased as Cd stress increased [20]. Moreover, in strawberries, the activities of SOD, CAT, and POD were significantly increased with higher Cd stress [21]. But in different species, the functions of these enzymes depend on different Cd stress concentrations. Moreover, under different Cd stress conditions on plants, chlorophyll content decreased significantly with increasing Cd stress [21]. Furthermore, cellular organelles deformed, disintegrated, and ultimately led to the death of plants [11,21]. 

In recent years, the remediation of heavy metal pollution has attracted considerable attention globally [22]. Therefore, to remediate metals, the topic of phytoremediation has become of great interest in many countries [22,23,24]. For the remediation of coastal Cd pollution, halophytes are more suitable than glycophytes [24]. Halophytes have higher growth rates and can maintain their continuous accumulation in toxic and saline environments [25]. Moreover, halophytes are also considered hyperaccumulators of Cd owing to their accumulation in vacuoles and cell walls [24,26]. In a higher saline condition, this plant can reduce toxic effects by lowering the Cd accumulation in the environment [27]. 

*S*. *portulacastrum* is a halophyte and is considered a phytoremediation species due to its higher ability to remove Cd from the Cd stress environment [24,27,28]. Numerous studies have suggested that the phytoremediation capacity of plants is related to their physiological responses under stressful environments [11,12,29]. Previous studies of Cd stress on *S*. *portulacastrum* were mostly related to growth, photosynthesis, fatty acid and lipid profiles, mineral nutrition, chemical form and subcellular distribution, and non-enzymatic responses [24,25,26,27,28,29]. These Cd stress studies were conducted with a narrow range of Cd concentrations. Moreover, other studies on *S*. *portulacastrum* suggested that higher salinity can reduce Cd toxicity by maintaining redox balance and water status, increasing potential Cd^2+^ chelators and reducing the amount of Cd accumulated in soluble fractions in the cell wall [27,30]. Uddin et al. (2020) [24] reported that under Cd stress conditions, *S*. *portulacastrum* can change Cd forms, compartmentation, and storage in vacuoles to reduce Cd toxicity. Therefore, higher Cd stress can drastically increase hydrogen peroxide production and membrane damage [28], but the mechanisms for reducing oxidative damage in *S*. *portulacastrum* are still unknown. To the best of our knowledge, there are no studies available on the physiological responses, especially the enzymatic response, under a wider range of Cd stress (50 μmol/L or μM to 600 μmol/L or μM of Cd concentrations). This higher Cd stress on *S*. *portulacastrum* can indicate tolerance mechanisms and adaptability in the severe Cd-polluted environment. Moreover, this physiological response mechanism also suggests the potential phytoremediation capacity in the Cd-polluted environment. 

The aims and objectives of this research are to examine the physiological responses, including MDA, SOD, CAT, and POD, of *S*. *portulacastrum* to hydroponically induced Cd stress. Moreover, this study also investigated the changes in chlorophyll content in leaves and the changes in the cellular ultrastructure of leaves and roots due to Cd stress. This study provides the basis for the restoration or remediation of alkali land, wetlands, coastal areas, and seawater contaminated by severe Cd pollution. Furthermore, this study also provides resistance mechanisms for *S*. *portulacastrum* in a severe Cd-polluted environment.

## 2. Results

### 2.1. Effect of Cd Stress on Chlorophyll Content of Leaves

In the analysis, it was found that the chlorophyll content of leaves was reduced with increasing Cd concentrations (Table 1).

**Figure 1:**
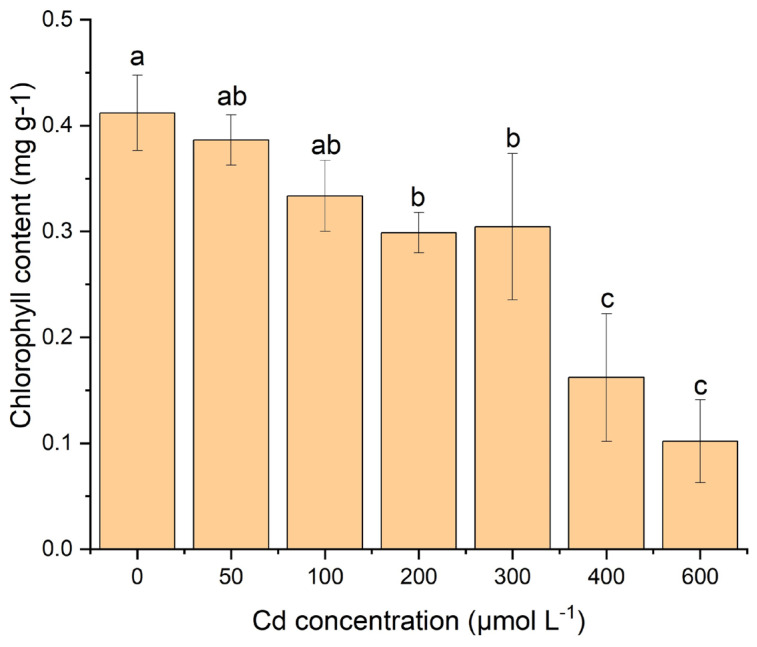
‘The variations of chlorophyll concentration (mg g^−1^) in leaves of *S*. *portulacastrum* under different Cd stress’. (According to ANOVA and Turkey’s test, distinct letters in various treatments indicate a significant difference (*p* < 0.05) between treatments).

There was a significant (*p* < 0.05) decrease in chlorophyll content in the concentration of 200 μM Cd with the control treatment (Figure 1). In addition, significantly (*p* < 0.05) decreased chlorophyll content was observed under the stress of 400 μM and 600 μM of Cd compared to all other treatments (Figure 1). For the stress of 600 μM Cd, the content of chlorophyll a, chlorophyll b, and total chlorophyll decreased by 81.75%, 56.10%, and 75.47%, respectively (Figure 1). The content of chlorophyll a was higher than chlorophyll b in leaves (Table 1). The ratio of chlorophyll a and b showed a similar trend of changes under Cd stress, and their ratio concentration was continuously decreased with increasing Cd stress (Table 1). Therefore, the ratio of chlorophyll a and chlorophyll b showed that the chlorophyll a concentration ratio was greater than chlorophyll b under the Cd stress (Figure 1).

### 2.2. Effect of Cd Stress on Lipid Peroxidation of Tissues

**Figure 2:**
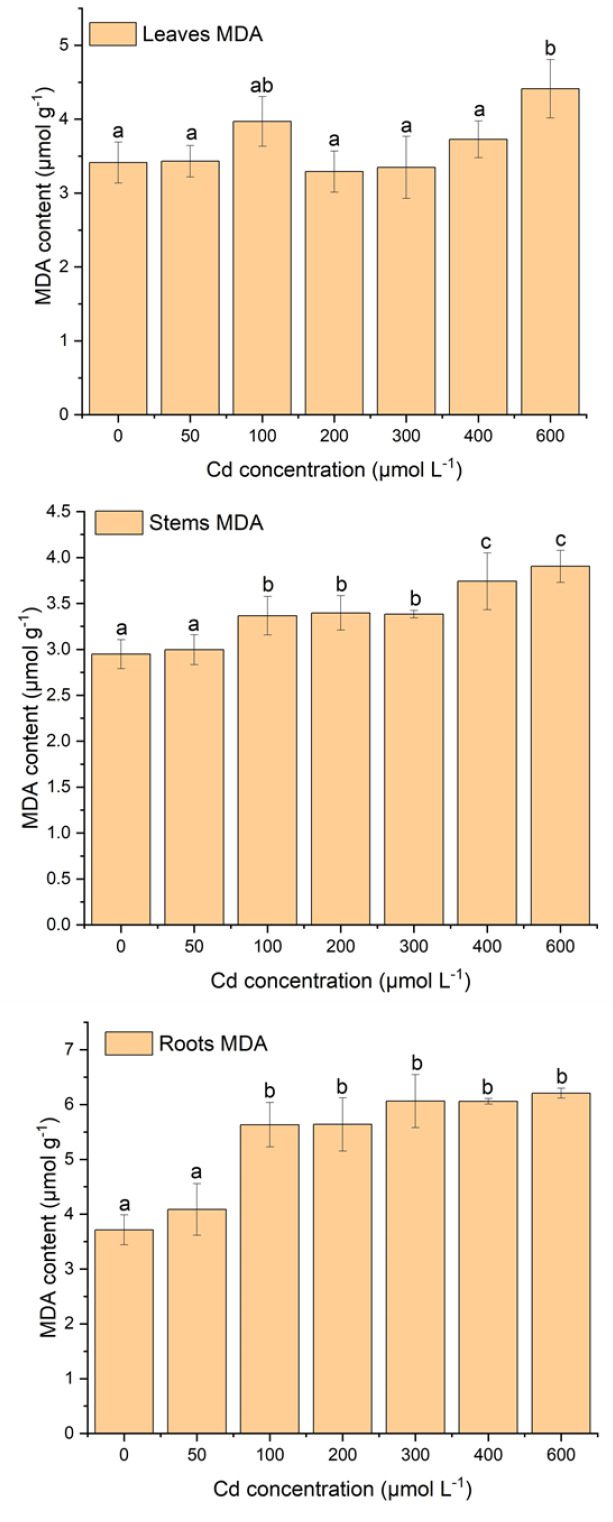
‘The variations of MDA content (µmol g^−1^) in leaves, stems, and roots of *S*. *portulacastrum* tissues under different Cd stress’. (According to ANOVA and Turkey’s test, distinct letters in various treatments indicate a significant difference (*p* < 0.05) between treatments).

The variations in MDA concentration in leaves, stems, and roots are displayed in Figure 2. The root tissues had higher MDA content than stems and leaves under different Cd stress conditions (Figure 2).

Stem had the lowest MDA content under the Cd stress. In leaves, MDA content showed a fluctuation in concentration with increasing Cd stress. At the concentration of 100 µM, there was a sharp increase in MDA but a decrease at 200 µM of Cd, and after that, there was a gradual increase in MDA concentration with increasing Cd stress (Figure 2). As a result, there is no significant difference between MDA content in leaves under different Cd stresses except for 600 µM of Cd treatment (Figure 2). In the stem and root, MDA concentrations increased gradually with increasing Cd stress. In stems, there was a significant (*p* < 0.05) difference in MDA content between the range of 0 µM to 50 µM and the range of 100 µM to 600 µM and between the range of 100 µM to 300 µM and the range of 400 µM to 600 µM of Cd stress. But in roots, there was no significant difference in MDA content between 100 and 600 µM of Cd stress, and only a significant difference was observed between 0 µM and 50 µM and 100 µM and 600 µM of Cd stress. In addition, MDA content was increased by 32.20% and 67.39% at 600 μM of Cd treatment relative to the control in the stem and root, respectively.

### 2.3. Effect of Cd Stress on Antioxidant System of S. portulacastrum

**Figure 3:**
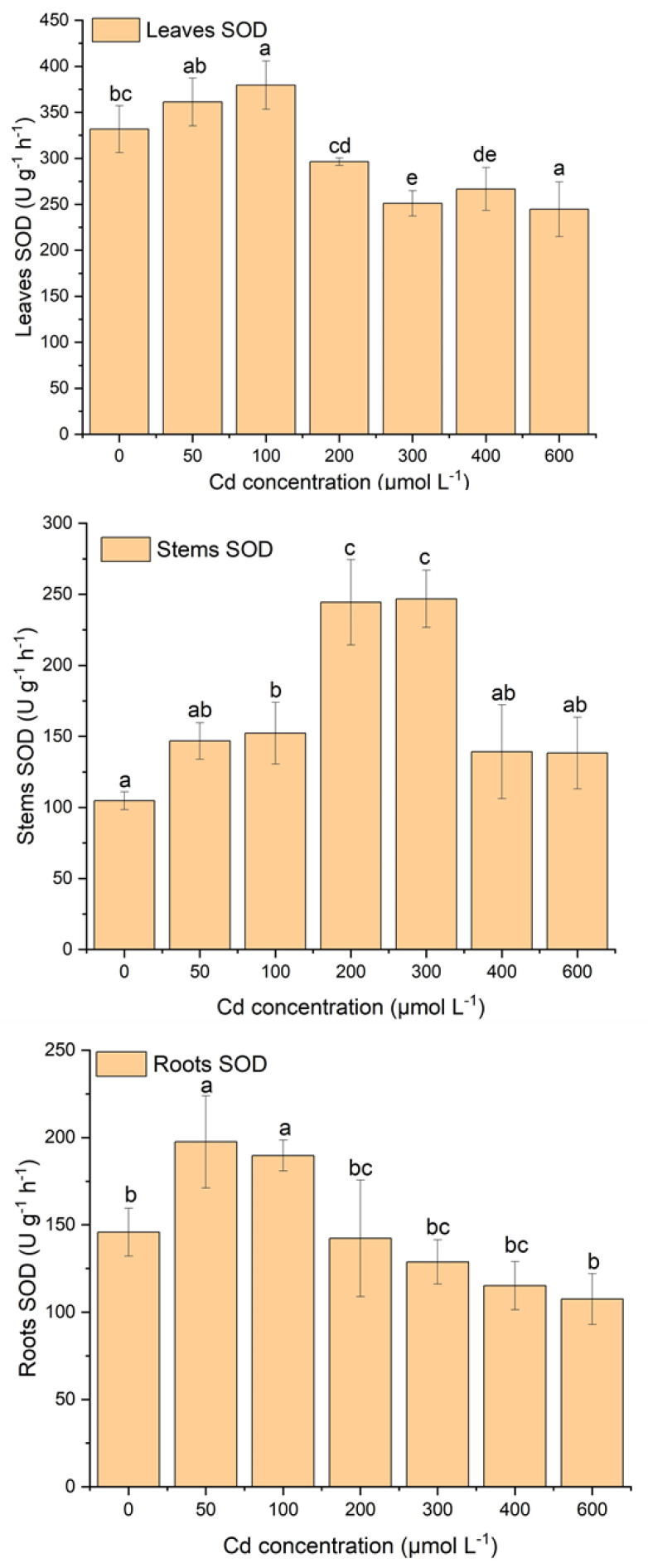
The variations in SOD activity in leaves, stems, and roots of *S*. *portulacastrum* tissues under different Cd stress. (According to ANOVA and Turkey’s test, distinct letters in various treatments indicate a significant difference (*p* < 0.05) between treatments).

**Figure 4:**
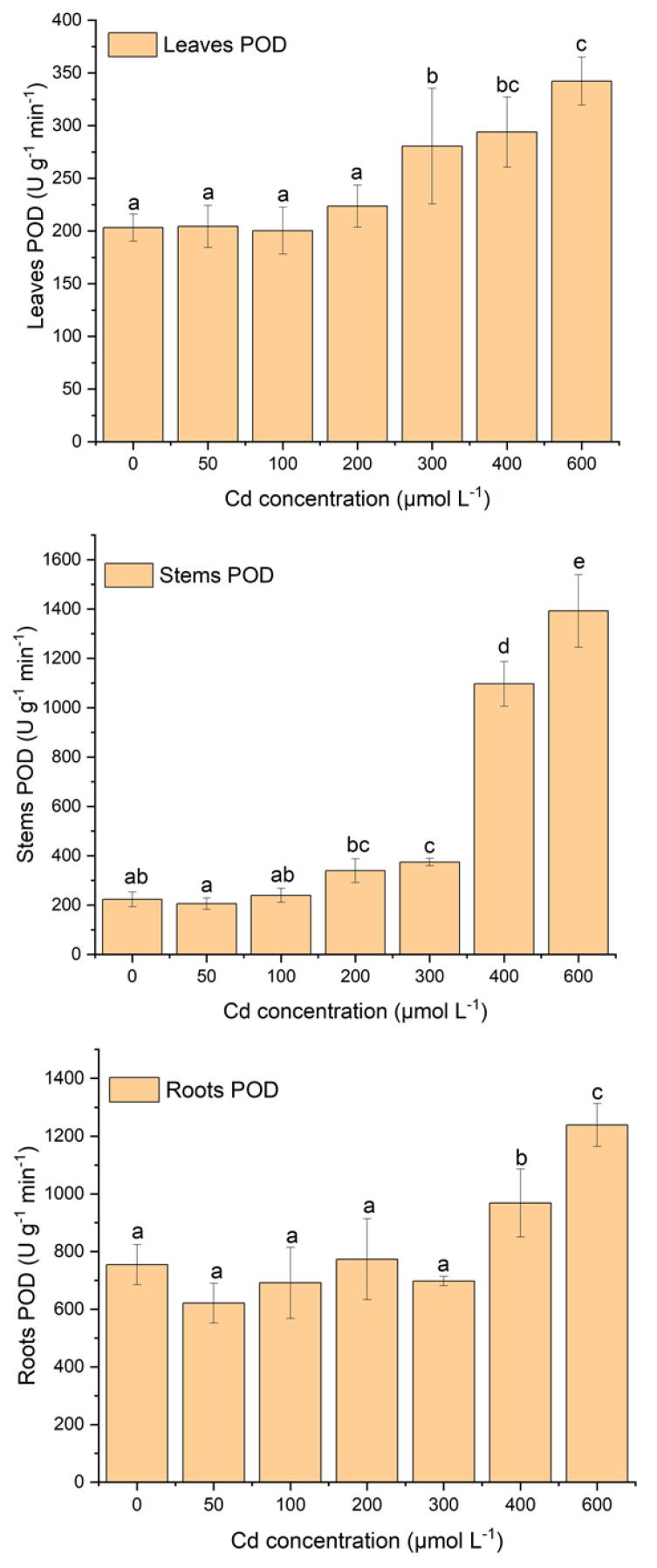
The variations in POD activity in leaves, stems, and roots of *S*. *portulacastrum* tissues under different Cd stress. (According to ANOVA and Turkey’s test, distinct letters in various treatments indicate a significant difference (*p* < 0.05) between treatments).

**Figure 5:**
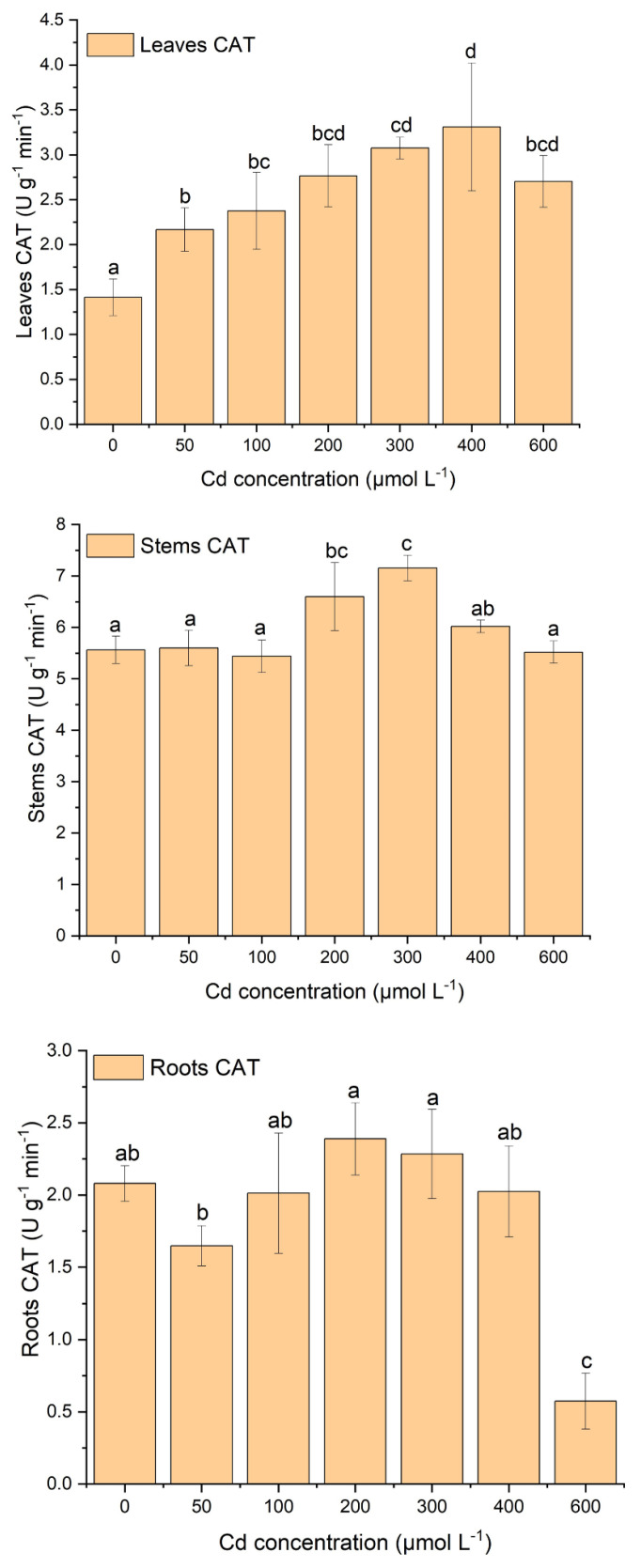
‘The variations of CAT activity in leaves, stems, and roots of *S*. *portulacastrum* tissues under different Cd stress’ (According to ANOVA and Turkey’s test, distinct letters in various treatments indicate a significant difference (*p* < 0.05) between treatments).

Figure 3, Figure 4 and Figure 5 display the variations in antioxidant enzyme activities such as CAT, SOD, and POD. Plant tissues (e.g., leaves, stems, and roots) showed different patterns of antioxidant enzyme activities with increasing Cd stress (Figure 3, Figure 4 and Figure 5). In leaves, SOD activity increased until 100 µM of Cd stress and then gradually decreased with increasing Cd concentration, but POD activity was relatively stable at 100 µM of Cd stress and then gradually increased with increasing Cd stress (Figure 3 and Figure 4). There was a significant decrease in SOD activity after 100 µM of Cd stress. Moreover, POD activity was also significantly (*p* < 0.05) higher after 200 µM of Cd stress. CAT activity showed gradually increasing concentrations with increasing Cd stress, except at 600 µM (Figure 5). Moreover, significantly (*p* < 0.05) higher CAT activity was observed between Cd stress concentrations and the control treatment (Figure 5).

In stems, SOD activity increased until 300 µM of Cd stress and then gradually decreased with increasing Cd stress (Figure 3). Moreover, under the stress of 200 µM and 300 µM of Cd, SOD activity was significantly (*p* < 0.05) higher than in other treatments. POD activity increased relatively slowly until 300 µM of Cd stress and then was sharply enhanced with proliferating Cd concentrations (Figure 4). In addition, in the treatment of 300 µM to 600 µM of Cd, POD activities increased significantly (*p* < 0.05) compared to other treatments. CAT activities were relatively stable at 100 µM of Cd stress, then sharply increased until 300 µM of Cd stress, and after that decreased with increasing Cd stress (Figure 5).

In roots, SOD and POD activities showed opposite behavioral patterns with increasing Cd stress treatments (Figure 3 and Figure 4). In terms of SOD activity patterns, they increase while POD activities decrease with increasing Cd stress treatments (Figure 3 and Figure 4). In the treatment with 50 µM and 100 µM of Cd, the activity of SOD was notably (*p* < 0.05) higher than in all other treatments with control (Figure 3). Furthermore, POD activity was significantly higher in the treatment of 400 µM and 600 µM Cd stress than in all other treatments (Figure 4). CAT activity did not follow any pattern but fluctuated with increasing Cd stress (Figure 5). The CAT activity was considerably (*p* < 0.05) lower at the treatment of 600 µM of Cd than all other treatments (Figure 5). Roots SOD and POD activities had opposite behavioral patterns; when SOD activity increased, POD activity decreased with increasing Cd treatments (Figure 3 and Figure 4).

### 2.4. Effect of Cd Stress on Ultrastructure of S. portulacastrum Cells

**Figure 6:**
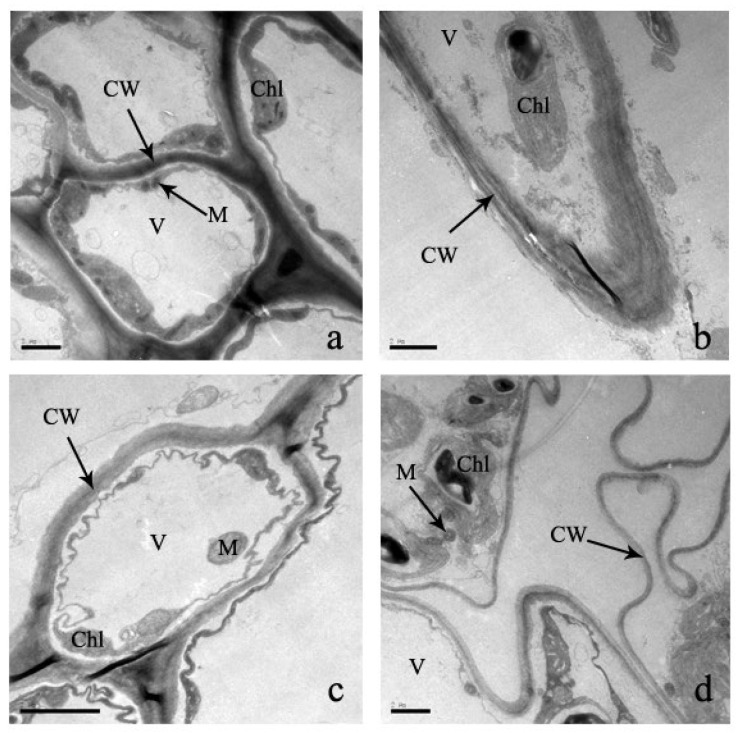
‘The effect of Cd stress (**a**): 0 μM control; (**b**): 100 μM Cd; (**c**): 300 μM Cd; (**d**): 600 μM Cd) on cells of *S*. *portulacastrum* leaves’ (CW: Cell wall, Chl: Chloroplast, V: Vacuole, and M: Mitochondrion).

Cells are the basic structural and functional units of biological life. In the control treatment, the leaf ultrastructure is smooth, as is the intake cell structure (Figure 6). In addition, cell organelles are arranged in order inside the cell wall, and mesophyll cells are also arranged (Figure 6). The inward folding of the plasma membrane is not obvious, and it contains few protrusions. Under the Cd stress, the leaf cell wall was distorted and thinner, and morphological deformation occurred (Figure 6). Moreover, mesophyll cells were folded inward, plasma membranes lost their shape and size, and cell cytoplasm was severally separated (Figure 6).

**Figure 7:**
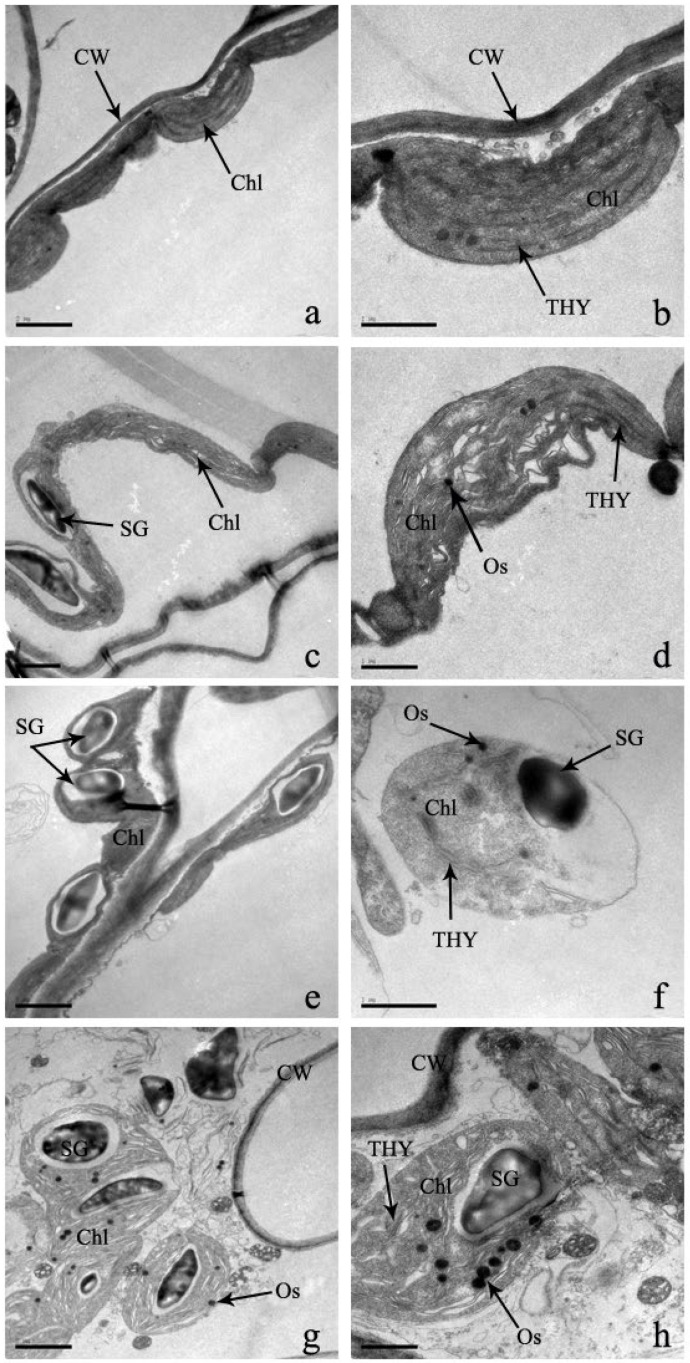
‘The effect of Cd stress ((**a**,**b**): 0 μM control; (**c**,**d**): 100 μM Cd; (**e**,**f**): 300 μM Cd; (**g**,**h**): 600 μM Cd) on chloroplast of *S*. *portulacastrum* leaves’ (CW: Cell wall, Chl: Chloroplast, THY: Thylakoids, SG: Starch granules, and Os: Osmiophilic granules).

The chloroplast is an important organelle of cells for the conversion of energy and nutrient manufacturing of plants for their normal life activities. There are significant changes in the chloroplast structure of Cd-stressed leaves compared to control leaves (Figure 7). The chloroplast of a leaf cell is mostly kidney-shaped, spindle-shaped, and arched-shaped (Figure 7). Under Cd stress, the shape of the chloroplast becomes irregular or nearly circular with proliferating Cd concentrations (Figure 7). In addition, the membrane of the outer borders of the chloroplast is gradually unclear, thylakoids disappear, and some vacuoles are formed in the thylakoids. Therefore, the stack of chloroplasts is chaotic, and the capsule is broken or disappears and disintegrates, changing the arrangement of chloroplasts completely (Figure 7). However, due to increased Cd stress, the distribution of changes gradually goes to the edges from the center of the cell and is randomly distributed in the cell. At concentrations of 300 μM and 600 μM Cd stress, the mesophyll cells are randomly distributed in the chloroplast and gradually larger. At the same time, the chloroplast is covered by starch granules.

**Figure 8:**
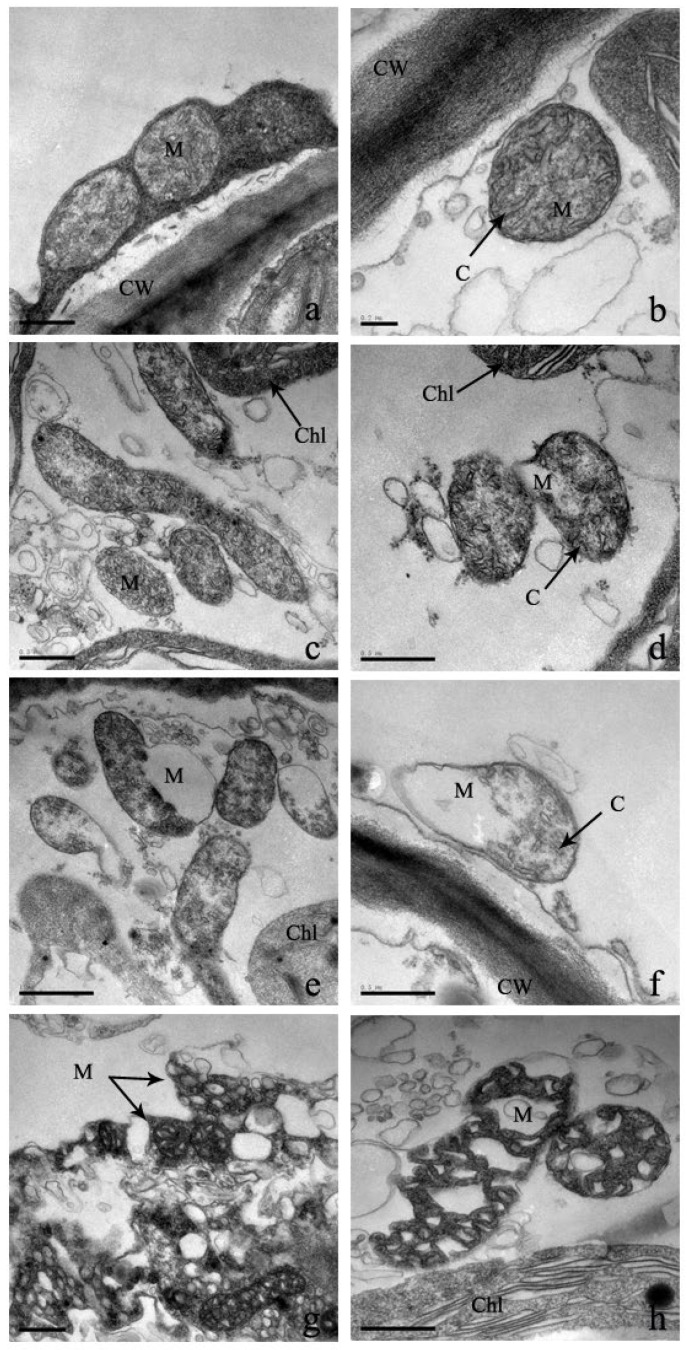
‘The effect of Cd stress ((**a**,**b**): 0 μM control; (**c**,**d**): 100 μM Cd; (**e**,**f**): 300 μM Cd; (**g**,**h**): 600 μM Cd) on mitochondria of *S*. *portulacastrum* leaves’ (CW: Cell wall, Chl: Chloroplast, M: Mitochondrion, and C: Cristae).

Mitochondria are the powerhouse of eukaryotes due to their ability to oxidize and release sugar, fats, and amino acids. Under low Cd stresses of 0 μM and 100 μM, the mitochondria of leaf cells are distributed along the cell membrane and adhere to the chloroplast (Figure 8). They are rod, globular, or elliptical and consist of a mitochondrial lumen and a clear outer layer. Under higher Cd stress, the distribution of mitochondria is gradually scattered, their shape is deformed, and the inner membrane is folded inwardly (Figure 8). In addition, when Cd stress reaches 600 μM, the outer membrane disappears and the mitochondria are severely lysed (Figure 8).

**Figure 9:**
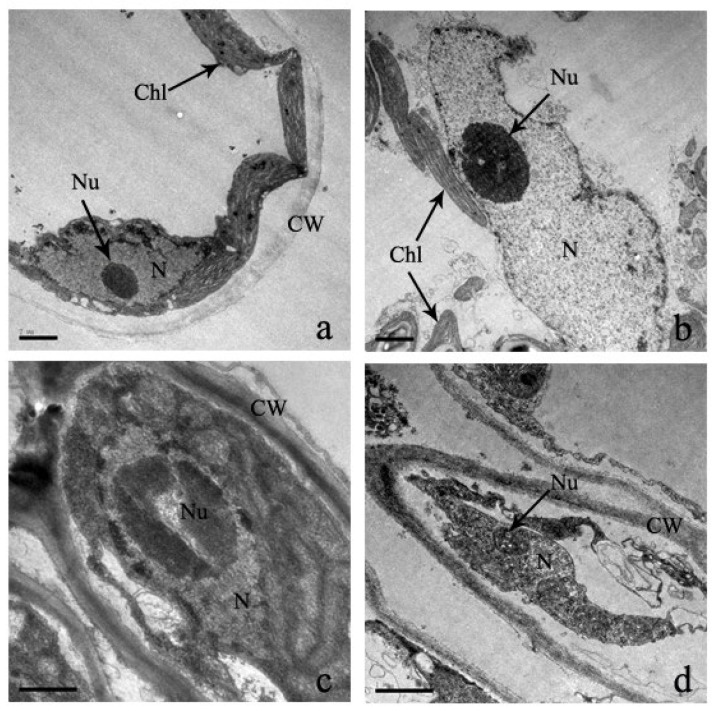
‘The effect of Cd stress ((**a**): 0 μM control; (**b**): 100 μM Cd; (**c**): 300 μM Cd; (**d**): 600 μM Cd) on nucleus of *S*. *portulacastrum* leaves’ (CW: Cell wall, Chl: Chloroplast, N: Nucleus, and Nu: Nucleolus).

In eukaryotic cells, the nucleus is the largest and most important organelle due to its central role in the regulation of cell genetics and metabolism. Moreover, it is the most significant marker that distinguishes eukaryotic cells from prokaryotic cells. In the control leaves, the nucleus is oval in shape, the nuclear membrane is clearly visible, and the location of the nucleolus is in the center of the cell nucleus (Figure 9). Under Cd stress, the nuclear membrane is distorted, resulting in the deformation of its shape, the migration and destruction of nucleoli outside of cells, the destruction of chromatin spills, and, finally, the reduction in nuclei by mass (Figure 9).

**Figure 10:**
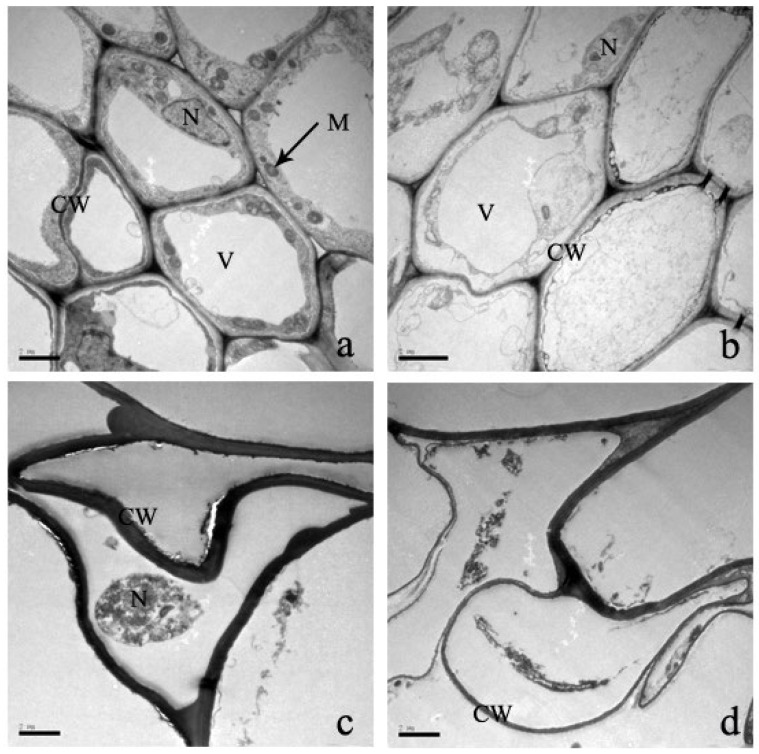
‘The effect of Cd stress ((**a**): 0 μM control; (**b**): 100 μM Cd; (**c**): 300 μM Cd; (**d**): 600 μM Cd) on cells of *S*. *portulacastrum* roots’ (CW: Cell wall, M: Mitochondrion, V: Vacuole, and N: Nucleus).

The root is the most valuable vegetative organ of plants and is responsible for the absorption of water, nutrients, and inorganic salts. In the control treatment, the ultrastructure of the root tip shows structural completeness with abundant cytoplasm and complete organelles (Figure 10). But with increasing Cd stress, root cell organelles are destroyed and the morphology is deformed, including cell wall breakdown, cell membrane protrusion, vacuoles constricting in the center of the cytoplasm, and plasmolysis (Figure 10).

**Figure 11:**
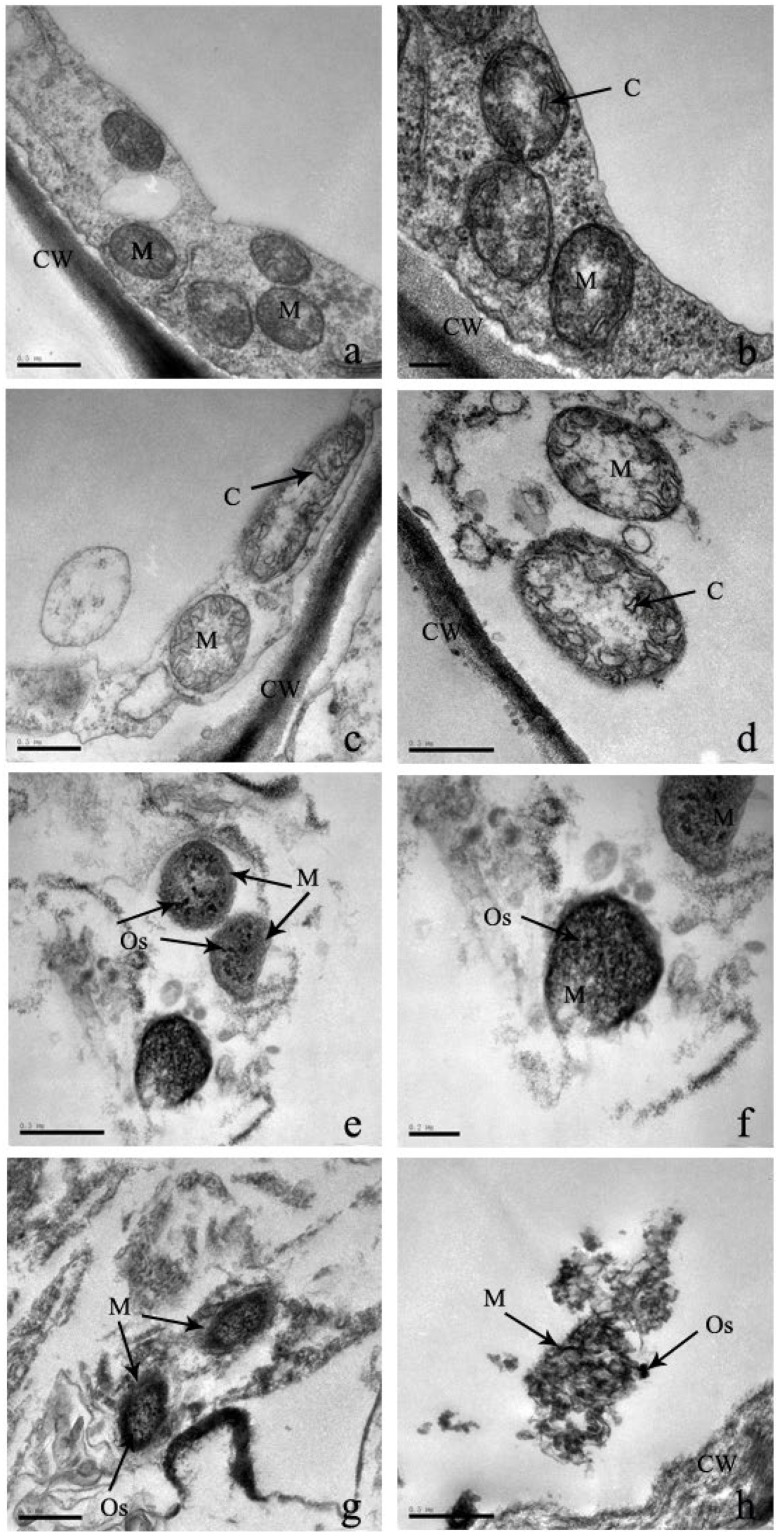
‘The effect of Cd stress ((**a**,**b**): 0 μM control; (**c**,**d**): 100 μM Cd; (**e**,**f**): 300 μM Cd; (**g**,**h**): 600 μM Cd) on mitochondrion of *S*. *portulacastrum* roots’ (CW: Cell wall, M: Mitochondrion, C: Cristae, and Os: Osmiophilic granules).

The mitochondria of the root cell are similar to those of leaf cell mitochondria, such as tubular condyles, dense mesenchymes, and clear membrane structures (Figure 11). Under the stress of 100 μM of Cd, the shape of the mitochondrion is deformed, with more openings but little overall effect on the structure (Figure 11). Under the stress of 300 μM, the mitochondrion becomes blurred with a more deformed structure. There is a significant increase in osmiophilic numbers at a concentration of 600 μM of Cd (Figure 11). Moreover, the membrane bilayer is completely dissolved, resulting in the entire mitochondria being on the verge of disintegration.

**Figure 12:**
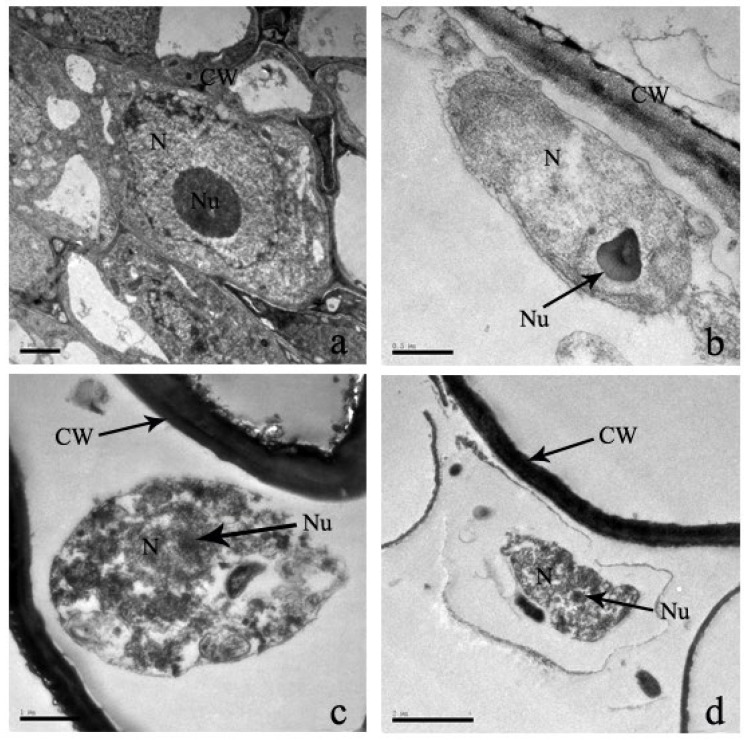
‘The effect of Cd stress ((**a**): 0 μM control; (**b**): 100 μM Cd; (**c**): 300 μM Cd; (**d**): 600 μM Cd) on nucleus of *S*. *portulacastrum* roots’ (CW: Cell wall, N: Nucleus, and Nu: Nucleolus).

The nuclear cell of root tissues shows an evenly distributed nucleus and a visible nuclear membrane, but after the treatment of 100 μM Cd stress, the nucleolus is slightly deformed, the nuclear membranes begin to dissolve, and the overall shape changes a little (Figure 12). In addition, increasing Cd stress shows that it completely dissolves the nuclear membrane, and nuclear materials diffuse into the cytoplasm and are scattered in the cytoplasm (Figure 12).

## 3. Discussion

### 3.1. Effect of Cadmium on Chlorophyll Content

Chlorophyll is an important component of the plant to maintain continuous food production through photosynthesis, which is responsible for its growth and reproduction. The ratio of chlorophyll a and b is an important parameter that reflects the senescence of plants and their photosynthetic efficiency [31,32,33]. The chlorophyll content of *S*. *portulacastrum* leaves gradually decreased with proliferating Cd concentrations (Table 1; Figure 1), suggesting the inhabitation of photosynthetic efficiency [30]. The decreased chlorophyll content may be due to the physiological and redox imbalances of plants due to higher Cd stress [30]. Therefore, higher Cd concentrations have an effect on the carotenoid contents of plants, resulting in an inhibitory effect on pigment and chromoenzyme biosynthesis, which is responsible for photosynthesis [21]. Numerous similar results have been reported previously in plant species such as *S*. *portulacastrum*, soybean seedlings, strawberries, *Potamogeton crispus*, and *Lepidium sativum* under Cd stress [21,30,34,35,36]. This result also indicates a higher decrease in chlorophyll a than chlorophyll b under increasing Cd stress. A comparable outcome was reported by Yang et al. (2011) [35], where 35.8% and 26.7% of chlorophyll a and chlorophyll b decreased with increasing Cd stress, respectively. There was no considerable effect of Cd stress on chlorophyll concentration under the stress of 200 μM of Cd, indicating that under this Cd concentration, plants can maintain their photosynthesis activity and accumulate nutrients from the nutrient solution or soils [30,36]. But after 400 μM to 600 μM of Cd stress, plants’ chlorophyll content significantly decreased (*p* < 0.05), suggesting a complete inhabiting of nutrients [37]. As a result, the senesce of plant leaves occurs due to the disruption of the chloroplast structure and the biosynthesis of pigments [30,38]. Overall, this result supports the idea that *S*. *portulacastrum* can maintain its photosynthetic activity under the stress of 200 μM of Cd, which could be a possible indicator of Cd tolerance in a polluted environment. 

### 3.2. Effect of Cadmium Stress on Lipid Peroxidation and Antioxidant Enzymes

MDA is a marker for plant lipid peroxidation, which can reveal oxidative damage and cell membrane integrity [17]. Heavy metal stress can stimulate plants to generate more reactive oxygen species (ROS), which can bind with cell organelles, cause lipid peroxidation, damage membranes, and disturb enzyme activation, reducing cell performance and viability [17,39,40]. In this study, MDA content was enhanced with the proliferation of Cd stress (Figure 2). Stem MDA content was significantly enhanced with increasing Cd stress, while stem and leaf MDA content were not significant. This result suggests that under Cd stress, stem cells are more damaged in terms of membrane destruction, enzyme inactivation, and performance than leaves and root cells [41]. The higher MDA content also indicates the destruction of redox balance under higher Cd stress [11,17,30]. Comparable results were reported in many plant species, such as Sassafras seedlings, *Amaranthus tricolor*, duckweed, *Ceratophyllum demersum*, and *Vigna radiata,* which had higher MDA content under increasing Cd stress [11,17,39,41,42]. Therefore, other abiotic stresses also caused a similar increase in MDA content in plants [43,44]. The MDA content of the root was higher than that of the leaves, which may be due to the root’s direct contact with the Cd solution, resulting in more severe Cd poisoning. Under the stress of 50 μM of Cd, plants did not produce MDA content in their tissues, indicating that under this stress environment, *S*. *portulacastrum* could maintain its redox balance and cell performance [30]. Several studies have reported that plants could reduce lipid peroxidation, cell membrane integrity, and redox balance by protecting chelators such as GSH, proline, and antioxidant enzymes [17,45,46].

Antioxidant enzymes can effectively reduce the oxidative stress of plants, and these enzymes can inhibit the Cd poisoning of plants [17]. In addition, antioxidant enzymes can break down superoxide radicals into oxygen and hydrogen peroxide, reducing the stress of heavy metals [17,47]. Under cadmium stress or a polluted environment, plants adjust the adverse effects through the adsorption of various response mechanisms. For *S*. *portulacastrum* to adapt in a Cd stress environment, response mechanisms include changes in chemical forms of Cd and their subcellular distribution, reduced adsorption of Cd, activity of antioxidant enzymes, etc. [24,27,28,30]. Antioxidant enzymes such as CAT, POD, and SOD are important detoxification mechanisms in plants to reduce cellular damage and ensure normal physiological activity under stressful environments [17,18].

In this study, there was a strong antioxidative response due to Cd stress in the leaves, roots, and stems of *S*. *portulacastrum* (Figure 3, Figure 4 and Figure 5). Similar findings were observed in many different plant species, such as *Acanthus ilicifolius*, Sassafras seedlings, *Ceratophyllum demersum,* etc. [11,17,39]. The activity of different antioxidant enzymes did not have similar concentration patterns with increasing Cd stress. SOD and CAT activity were increased in a certain Cd stress; after that, they decreased with increasing Cd stress, but POD activity enhanced with proliferating Cd stress (Figure 3, Figure 4 and Figure 5). These findings are comparable to those of other studies with different species of plants [11,17].

These results suggested that three antioxidant enzymes were working concurrently to reduce reactive oxygen species from different plant parts of *S*. *portulacastrum* [11,39]. These findings also suggested that POD was likely to be the main antioxidant enzyme activity for protection from the reactive oxygen species [11]. Moreover, CAT had likely higher removal activity than SOD, indicating that CAT was one of the higher supportive antioxidants with POD. These results also indicated that in a stress environment, *S*. *portulacastrum* could enhance the POD and CAT enzyme activity to enhance tolerance in a Cd-polluted environment [11]. A similar result was reported in Sassafras seedlings [11]. It was found that SOD activity increased at 100 μM of Cd stress and then gradually reduced with exacerbating Cd stress, but at the same time, CAT activity increased until 300 μM of Cd stress and then reduced after higher stress. But POD had lower activity until 100 μM, and after that, it gradually reduced with exacerbating Cd stress. These results suggest that SOD was the dominant detoxification enzyme activity in a low-stress environment (100 μM of Cd) [11]. Moreover, in the stress range of 100 μM to 300 μM, CAT activity was predominant, but in the higher stress range, POD played a dominant role in the tolerance of higher Cd stress. This study also suggested the highest activity of SOD for leaves, CAT for stems, and POD for roots (Figure 3, Figure 4 and Figure 5). These variations in antioxidants may be related to the different contents of ROS accumulated in different parts of plants. These variations could possibly be related to different gene expressions in different sites of the plants [7]. This needs to be further studied in the future. Therefore, under the stress condition, non-enzymatic antioxidants such as proline and glutathione may increase their activity in protecting plants from accumulating ROS in their tissues [48]. This may be possibly another reason for the variation in their antioxidant enzymes under stress conditions [48]. This study did not investigate non-enzymatic antioxidant responses. Several studies suggest that non-enzymatic antioxidants, including proline, ascorbic acid, tocopherols, glutathione, phenolics, flavonoids, and carotenoids, play an important role during the plant physiological process under stress conditions [40,48,49]. These non-enzymatic activities and their interactions with enzymatic responses should be taken into account in the future to identify the comprehensible tolerance and resistance mechanisms of *S*. *portulacastrum* in a polluted environment.

### 3.3. Effect of Cadmium Stress on Ultrastructure of Cells

In the plant’s physiological process, the cellular structure and functions are complementary [24]. As a result, the effect of heavy metal stress on the cellular ultrastructure of leaves and roots is very important for understanding the physiological changes and functional effects [50]. In this experiment, under increasing Cd treatments, cell walls became gradually thin and curved, fractures were distorted, and the shape of each organelle was deformed and ultimately disintegrated. Each starch granule became larger, and granules increased in number. Stress in plants is indicated by an increase in starch granules [14,50]. The chloroplast and mitochondrial structures were greatly damaged, which has effects on the photosynthesis of *S*. *portulacastrum*. As a result, the energy supply was seriously damaged, which hindered the growth and physiological metabolism of *S*. *portulacastrum*. Similar chloroplast and mitochondrion alternations were observed in other plant species [14,50]. Another very important alternation was the increase in nuclei in the cell nucleus, which can increase protein synthesis and antioxidant enzymes, which can enhance the synthesis of Cd-complexed proteins for reducing the damage to plants [14]. Similar ultrastructural alternations were found in different plant species under Cd stress, such as *Juncus effusus* L., *Oryza sativa* L., and Miscanthus [50,51,52]. Moreover, the cell structure of these species was severely altered under the treatment of 100 μM of Cd stress [50,51,52]. But under 100 μM of Cd stress, the ultracellular structure of *S*. *portulacastrum* was not seriously damaged, indicating stronger tolerance to a Cd-polluted environment.

## 4. Materials and Methods

### 4.1. Experimental Plant and Treatments Protocol

*S*. *portulacastrum* seedlings were procured from the base station in Quanzhou. After that, fragments were sterilized for 5 min with a calcium hypochlorite solution, and distilled water was used to clean them [24]. Then, fragments were raised in a greenhouse using 20 parts per thousand salinity and Hoagland nutrient conditions [24]. From the rearing plants, 63 fragments were selected for experimental treatment at concentrations of 0 μmol/L or 0 μM (control) to 600 μmol/L or 600 μM of Cd for 28 days. The experimental treatments and protocols were acquired from Uddin et al. 2020 [24]. 

For this experiment, 2.5 L of the Hoagland nutrient was placed into a plastic pot (17 cm by 14 cm) with a pH of 6.5. Each treatment pot had three pieces of plant fragment, and the fragment plants were raised in a floating bed. The growth parameters were a 16 h light period with 250 µmol m^2^ S^−1^ of light intensity, 25 °C temperature, and 60% to 80% relative humidity [24]. There were three replicates of each treatment.

### 4.2. Determination of Chlorophyll Content in Leaves of S. portulacastrum

In this experiment, the N, N’-dimethylformamide (DMF) method was employed to evaluate the amount of chlorophyll in plant leaves [53,54]. The DMF extraction method is convenient, stable, and provides a more accurate or complete measurement of chlorophyll content [35]. For this experiment, fresh, healthy, same-colored, and same-size leaves were collected from *S*. *portulacastrum*. Leaves were sliced into tiny pieces [55]. Then, 0.2 g of cutting leaves were placed into a tube, and 10 mL of DMF extraction was added [55]. The tube was put into a horizontal shaker for 24 h. After that, extracts from the tube were collected in 1 cm cuvettes for the quantification of chlorophyll content. A visible spectrophotometer was used to quantify chlorophyll content under the absorbance of 647 nm to 664.5 nm [55].

### 4.3. Determination of MDA Content in Tissues of S. portulacastrum

Malondialdehyde (MDA) is an important parameter that indicates the level of lipid peroxidation in plant tissues [56]. In this experiment, the thiobarbituric acid method was used to quantify the MDA content in plant tissues [16]. Fresh plant tissue (1 g) was placed into a centrifuge tube, 10% trichloroacetic acid in 10 mL was added, and the tube was centrifuged for 10 min at 12,000× *g* [16]. After that, 2 mL of clear supernatant was collected from the centrifuging sample, and 10% trichloroacetic acid was added to dissolve 2 mL of 0.6% thiobarbituric acid [16]. Then, this reaction solution was heated up for 20 min in boiling water [16]. The solution was cooled and centrifuged for 12 min at 12,000× *g* [16]. A GENESYS 10S UV-Visible (Thermo Fisher Scientific, Waltham, MA, USA) type spectrophotometer was used to determine the absorbance at 450, 532, and 600 nm, respectively [57].

### 4.4. Determination of the Activities of Antioxidant Enzymes in S. portulacastrum

In a contaminated and stressed environment, plants’ antioxidant enzymes can reduce reactive oxygen species by regulating enzyme activity to reduce plant damage [58]. The antioxidant enzymes that were investigated were catalase (CAT), peroxidase (POD), and superoxide dismutase (SOD). The activity of these antioxidant enzymes was determined by using a spectrophotometer. Fresh tissues (e.g., leaves, stem, and root) of 0.5 g were placed into a grinding bowl with 2 mL of 0.05 mol L^−1^ ‘phosphate buffer solution’ at pH 7.8; for rapid grinding, 1% polyvinylpyrrolidone (PVP) was added; and the mixture was placed in a freezing bath [17]. Then, the sample was centrifuged at 4 °C for 15 min at 10,000× *g*/min and used as a test sample for SOD, POD, and CAT analysis [17]. For the measurement of SOD activity, the ‘nitrogen blue tetrazolium’ (“NBT method”) photochemical reduction method was used. For the measurement of POD, the Guaiacol oxidation method was used [59]. With this technique, a 470 nm wavelength was used to measure increasing absorbance [17,38]. Moreover, the decrease in absorbance at 240 nm was used to quantify CAT activity, which resulted in consuming the substrate H_2_O_2_ [17,59].

### 4.5. Determination of Cell Structures and Their Deformations of S. portulacastrum

For this experiment, fresh young leaves (3rd from the tip) and tender roots were collected from the *S*. *portulacastrum* plant samples. In this experiment, the Cd stresses of 0 μmol/L or 0 μM, 100 μmol/L or 100 μM, 300 μmol/L or 300 μM, and 600 μmol/L or 600 μM were used. After that, samples were gently washed and dried to remove surface moisture. Leaves were cut into 1 mm^2^ of tiny pieces and immediately put into glutaraldehyde (‘2.5%, *v*/*v*, EM grade, Merck, Darmstadt, Germany’) in a 0.1 M ‘phosphate buffer solution’ (PBS) for 2 h with a pH of 7.4 [30]. After that, samples were rinsed every 15 min with PBS three times, fixed with 1% (*w*/*v*) osmium tetroxide for 2 h, and washed four times with Milli-Q water and acetone for sequential dehydration [30]. All of the treatments were conducted at 4 °C. After that, samples were set into resin and sliced with the ‘Leica ultra-cut UCT microtome’ (‘Leica Microsystems, Wetzlar, Germany’) [30]. A diamond knife was used to cut samples with an ultra-thin section (70 nm in thickness). These sections were set up on copper grids with a mesh size of 200 and were uncoated. For contrasting these sections, the solutions of ‘conventional uranyl acetate’ (30 min) and ‘Reynolds lead citrate’ (5 min) were used. [30]. Using a ‘Gatan Ultrascan ES1000 CCD’ camera and a ‘Transmission Electron Microscope’, samples of sections were examined.

### 4.6. Statistical Analysis

An analysis of variance (one-way) was conducted to differentiate between Cd treatments on *S*. *portulacastrum*. To evaluate the significant difference among treatments, Turkey’s test was used [24]. Analyses were performed using SPSS 22.0 (SPSS for Windows, SPSS Inc., Chicago, IL, USA).

## 5. Conclusions

This study investigated the physiological responses, including changes in chlorophyll content, changes in MDA content, changes in antioxidant enzyme activity, and cellular ultrastructural changes, of *S*. *portulacastrum* under Cd stress. Chlorophyll content decreased under Cd stress conditions, which can reduce the synthesis of chlorophyll and thus affect the photosynthetic efficiency of plants. MDA content increased under Cd stress in leaves, stems, and roots, indicating the accumulation of ROS, which causes membrane lipid peroxidation. Cd stress can enhance the antioxidant enzyme activity, and their activities fluctuate with proliferating Cd stress. SOD was the dominant antioxidant activity under the stress of 100 μM, while POD was the dominant activity under higher Cd (>100 μM) stress. The ultracellular structure of leaf and root cells was seriously damaged, such as by cell wall thinning, mitochondria and nuclei deformation, disintegration, larger starch granules, etc., due to Cd stress. Moreover, under higher Cd stress conditions, cells can increase the number of nucleolus, which can increase the synthesis of new proteins, and this mechanism may be responsible for the possible detoxification or tolerance of Cd in a polluted environment. Overall, serious cellular structural damage in *S*. *portulacastrum* was observed at 300 μM of cadmium, suggesting stronger tolerance in the Cd-contaminated environment.

## Figures and Tables

**Table 1:** The variations of chlorophyll content (average ± SD mg g^−1^) in leaves of *S*. *portulacastrum* under different Cd stress.

Cd Concentration (μM)	Chl a (mg g^−1^)	Chl b (mg g^−1^)	Chl(a + b) (mg g^−1^)	Chl a/b
0	6.52 ± 0.57	2.05 ± 0.17	8.56 ± 0.74	3.18 ± 0.06
50	5.93 ± 0.37	1.99 ± 0.01	7.92 ± 0.38	2.98 ± 0.16
100	5.07 ± 0.52	1.78 ± 0.13	6.85 ± 0.65	2.84 ± 0.10
200	4.62 ± 0.22	1.61 ± 0.17	6.23 ± 0.39	2.88 ± 0.16
300	3.94 ± 0.20	1.45 ± 0.00	5.39 ± 0.20	2.81 ± 0.19
400	2.24 ± 0.50	1.11 ± 0.11	3.35 ± 0.61	1.95 ± 0.53
600	1.19 ± 0.63	0.90 ± 0.15	2.10 ± 0.78	1.27 ± 0.45

SD = Standard Deviation.

## Data Availability

The dataset used and analyzed during the present study are available from corresponding author upon reasonable request.
